# Supplementary material for: Keratinocytes mediate innocuous and noxious touch via ATP-P2X4 signaling
Source: eLife. 2018 Jan 16;7:e31684. doi: 10.7554/eLife.31684 (PMC5777822; doi:10.7554/eLife.31684)
Supplement: Transparent reporting form [file elife-31684-transrepform.docx]

***eLife’s* transparent reporting form**

We encourage authors to provide detailed information *within their submission* to facilitate the interpretation and replication of experiments. Authors can upload supporting documentation to indicate the use of appropriate reporting guidelines for health-related research (see [EQUATOR Network](http://www.equator-network.org/%20)), life science research (see the [BioSharing Information Resource](https://biosharing.org/)), or the [ARRIVE guidelines](http://www.plosbiology.org/article/info:doi/10.1371/journal.pbio.1000412) for reporting work involving animal research. Where applicable, authors should refer to any relevant reporting standards documents in this form.

If you have any questions, please consult our Journal Policies and/or contact us: [editorial@elifesciences.org](mailto:editorial@elifesciences.org).

**Sample-size estimation**

- You should state whether an appropriate sample size was computed when the study was being designed
- You should state the statistical method of sample size computation and any required assumptions
- If no explicit power analysis was used, you should describe how you decided what sample (replicate) size (number) to use

Please outline where this information can be found within the submission (e.g., sections or figure legends), or explain why this information doesn’t apply to your submission:

We did not perform any a-priori estimation of sample sizes. However, we followed the standards in the field and our prior findings for n’s per group closely, and, accordingly, used 4 – 15 individual biological replicates for each data point. An n of at least 4 animals was used for patch clamp experiments adding up to 20-40 cells. For behavior experiments no less than 8 animals were in each group. Skin nerve recordings were made from at least 20 animals for each fiber type. ATP release measurements using ATP biosensors were made from 4 animals and 8 sections of skin in total, where multiple locations of the skin were stimulated. With these numbers of replicates we were able to detect differences between means that were in the order of 1 to 2 standard deviations. We have consulted with statistician Aniko Szabo, Ph.D. in Biostatistics for our sample sizes and statistical analyses.

**Replicates**

- You should report how often each experiment was performed
- You should include a definition of biological versus technical replication
- The data obtained should be provided and sufficient information should be provided to indicate the number of independent biological and/or technical replicates
- If you encountered any outliers, you should describe how these were handled
- Criteria for exclusion/inclusion of data should be clearly stated
- High-throughput sequence data should be uploaded before submission, with a private link for reviewers provided (these are available from both GEO and ArrayExpress)

Please outline where this information can be found within the submission (e.g., sections or figure legends), or explain why this information doesn’t apply to your submission:

Biological replicate numbers have been provided for all experiments in the figure legends. N’s on the graph for behavior are the actual number of animals, and for electrophysiological experiments the n’s reported on the graph are the number of cells or afferent fibers. No outliers were encountered in any of the experiments.

**Statistical reporting**

- Statistical analysis methods should be described and justified
- Raw data should be presented in figures whenever informative to do so (typically when N per group is less than 10)
- For each experiment, you should identify the statistical tests used, exact values of N, definitions of center, methods of multiple test correction, and dispersion and precision measures (e.g., mean, median, SD, SEM, confidence intervals; and, for the major substantive results, a measure of effect size (e.g., Pearson's r, Cohen's d)
- Report exact p-values wherever possible alongside the summary statistics and 95% confidence intervals. These should be reported for all key questions and not only when the p-value is less than 0.05.

Please outline where this information can be found within the submission (e.g., sections or figure legends), or explain why this information doesn’t apply to your submission:

Statistical tests are described in the “statistical analysis” methods sub-section page 40 and 41 line 988- 1008. Test specifics are described in the corresponding figure legend for each experiment. Mechanical thresholds and conduction velocities of C fibers are reported on page 12 line 289-291 and for Aδ and Aβ fibers on page 12 line 297-303 in the results section. For *Advillin-P2rx4* studies, conduction velocities of all fibers are reported in the results section on page 17 and 18 line 436-440. We have consulted with statistician Aniko Szabo, Ph.D. in Biostatistics for our statistical analyses.

(For large datasets, or papers with a very large number of statistical tests, you may upload a single table file with tests, Ns, etc., with reference to sections in the manuscript.)

**Group allocation**

- Indicate how samples were allocated into experimental groups (in the case of clinical studies, please specify allocation to treatment method); if randomization was used, please also state if restricted randomization was applied
- Indicate if masking was used during group allocation, data collection and/or data analysis

Please outline where this information can be found within the submission (e.g., sections or figure legends), or explain why this information doesn’t apply to your submission:

For all assays, animals were randomly assigned to the different treatment groups, where the experimenter was blinded to both genotype and treatment given page 29 line 700-702, page 30 line 729-730 and line 741-742, page 32 and 33 line 796-804, and page 34 line 827-828.

**Additional data files (“source data”)**

- We encourage you to upload relevant additional data files, such as numerical data that are represented as a graph in a figure, or as a summary table
- Where provided, these should be in the most useful format, and they can be uploaded as “Source data” files linked to a main figure or table
- Include model definition files including the full list of parameters used
- Include code used for data analysis (e.g., R, MatLab)
- Avoid stating that data files are “available upon request”

Please indicate the figures or tables for which source data files have been provided:
